# EGFR kinase domain mutation positive lung cancers are sensitive to intrapleural perfusion with hyperthermic chemotherapy (IPHC) complete treatment

**DOI:** 10.18632/oncotarget.6491

**Published:** 2015-12-08

**Authors:** Hongjuan Zhang, Cheng Zhan, Ji Ke, Zhiqiang Xue, Aiqun Zhang, Kaifeng Xu, Zhirong Shen, Lei Yu, Liang Chen

**Affiliations:** ^1^ School of Life Science, Tsinghua University, Beijing 100084, China; ^2^ National Institute of Biological Sciences, Beijing 102206, China; ^3^ Beijing Tongren Hospital, Capital Medical University, Beijing 100730, China; ^4^ The General Hospital of People's Liberation Army (301 Hospital), Beijing 100853, China; ^5^ Peking Union Medical College Hospital, Beijing 100730, China; ^6^ Collaborative Innovation Center of Systems Biomedicine, Shanghai Jiao Tong University School of Medicine, Shanghai 200240, China; ^7^ National Institute of Biological Sciences, Collaborative Innovation Center for Cancer Medicine, Beijing, 102206, China

**Keywords:** EGFR, hyperthermic chemotherapy, lung cancer, kinase domain mutation

## Abstract

Lung cancer is the global leading cause of cancer-related deaths. A significant portion of lung cancer patients harbor kinase domain mutations in the epidermal growth factor receptor (EGFR). While EGFR tyrosine kinase inhibitors (TKI) effectively shrink tumors harboring mutant EGFR, clinical efficacy is limited by the development of TKI resistance. Effective alternatives are desperately needed in clinic for treating EGFR kinase domain mutation positive lung cancer. In our clinic in treating M1a lung cancer patients through intrapleural perfusion with hyperthermic chemotherapy (IPHC) followed by cycles of systemic chemotherapy (we termed this procedure IPHC complete treatment, IPHC-CT), we found dramatic tumor shrinkage in mutant EGFR-positive patients. We further confirmed the sensitivity of EGFR mutation-positive lung cancer cell lines derived from patients to HC (hyperthermic chemotherapy) treatment. We found that hyperthermia promoted accumulation of cisplatin in lung cancer cells. Hyperthermia and cisplatin synergistically downregulated the EGFR protein level, leading to quenching of signal from EGFR and induction of apoptosis. Our work therefore showed IPHC-CT is an effective treatment for EGFR kinase domain mutation positive lung cancer patients.

## INTRODUCTION

Non-small cell lung cancer (NSCLC), consisting of lung adenocarcinoma, squamous cell carcinoma, and large cell carcinoma, is the major pathological type of lung cancer. Currently, Lung cancer is the leading cause of cancer-related deaths with an estimated 1.38 million deaths as of 2008 [1–3]. Chemotherapy remains the mainstay treatment in advanced disease and shows marginal efficacy.

A significant portion of lung cancer patients harbor kinase domain mutations in the epidermal growth factor receptor (EGFR) [4, 5] which sensitizes lung cancer cells to EGFR tyrosine kinase inhibitors (TKI) [6, 7]. Remarkably rapid and often profound responses to EGFR TKIs, such as gefitinib or erlotinib, are frequently observed in a portion of lung cancer patients, in particular, non-smoker Asian females with pulmonary adenocarcinoma. Unfortunately, clinical efficacy is limited by the development of TKI resistance. An EGFR secondary mutation, T790M, is the most frequent cause of TKI resistance in patients [8, 9]. Several alternative strategies are now in various phases of clinical trials, including certain second-generation [10] and third-generation of irreversible inhibitors [11–13]. Data showed limited success of second-generation inhibitors, while the clinical performance of third-generation inhibitors remains to be determined. Currently, an effective alternative therapy is desperately needed for lung cancer patients positive for EGFR kinase domain mutations.

By irrigating the pleural space for 2 hours with hot saline solution containing cisplatin using specially devised extracorporeal circuits, this so-called intrapleural perfusion with hyperthermic chemotherapy (IPHC) achieved significantly longer overall survival [14]. However, biomarkers capable of predicting a specific population most likely to benefit from IPHC treatment remain to be determined.

Here, we report our clinical data to show that EGFR kinase domain mutation-positive NSCLC is associated with dramatic regression of patient's tumor to IPHC followed by cycles of systemic chemotherapy (IPHC-CT) and longer overall survival. We also show that hyperthermic chemotherapy is highly effective in killing mutant EGFR-driven lung cancer cells. EGFR mutation positive lung cancer cell lines showed enhanced ability to accumulate cisplatin in cell during hyperthermic treatment. In addition, 42°C (hyperthermia) and cisplatin (chemotherapy) synergistically downregulate the EGFR protein levels and inhibit EGFR signaling, leading to the activation of apoptotic pathways. Our work illustrates that IPHC-CT should be recommended for EGFR mutation positive lung cancer patients.

## RESULTS

### Patients positive for EGFR kinase domain mutation benefited from IPHC-CT treatment

**Figure 1:**
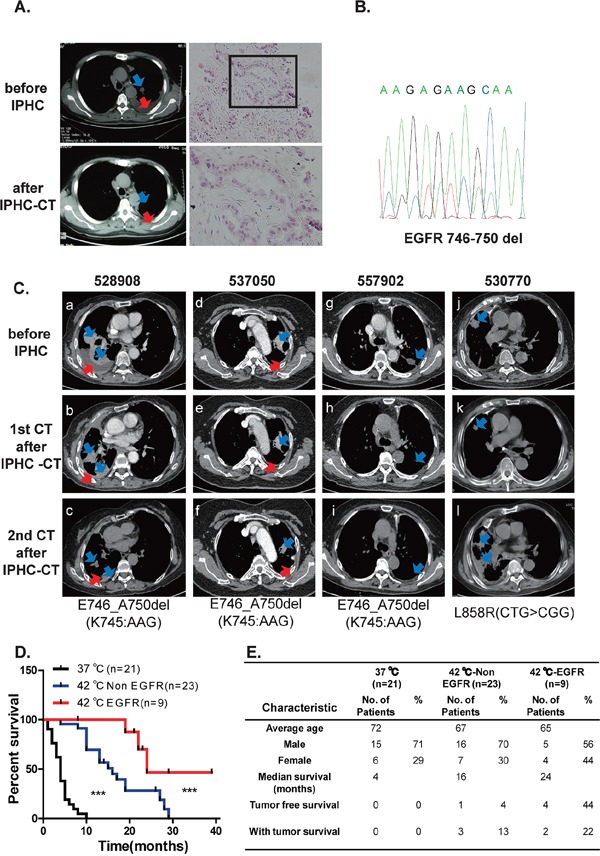
Lung cancers positive for EGFR mutation are sensitive to IPHC-CT treatment **A.** Tumor nodule in left lobe shrank in response to IPHC-CT treatment (pre-treatment shown in left upper panel, post-treatment shown in left lower panel, tumor highlighted with blue arrow-head, pleural effusion highlighted with red arrow-head). H&E staining of needle biopsy confirmed adenocarcinoma lung cancer in the patient#517945 (right panel). **B.** Sanger sequencing confirmed exon19 deletion mutation in EGFR. **C.** 4 patients (numbered 528908, 537050, 557902 and 530770) are diagnosed with lung cancer adenocarcinoma. EGFR mutations were detected in the tumor tissues. All patients were treated through IPHC-CT. CT images were listed to show before and after first and second time of CT scan after IPHC-CT treatment, EGFR mutation status was listed below. (a, b, c for 528908, d, e, f for 537050, g, h, i for 557902 and j, k, l for 530770 are the CT images for each patient in the order of time specified in the Supplementary patients summary). **D.** Comparison of survival rate among three groups (chemotherapy under 37°C, IPHC-CT against non-EGFR mutation and IPHC-CT against EGFR mutation patients). **E.** Baseline characteristics and survival rate of the 3 groups.

Hyperthermic chemotherapy has been repetitively confirmed to show efficacy in lung cancer patients [14–16]. However, predictive biomarkers of responsive patients remain unexplored. We have been treating patients with lung cancer of M1a and notice that a portion of patients respond dramatically to IPHC-CT. A typical case was patient# 517945 (male, 54 years old). The patient reported to hospital due to chest tightness and suffocation in 2009. Lung CT examination revealed multiple mediastinal lymph nodes and a nodule in left upper lobe (size: 24 mm × 18 mm) with the appearance of lobular contour and spiculated margin (Figure 1A, upper left panels, highlighted with blue arrow-head), and left pleural effusion (Figure 1A, upper left panels, highlighted with red arrow-head). CT guided needle biopsy confirmed lung adenocarcinoma in September 2009 (Figure 1A, upper right and lower right panels for high- and low-magnitude microscopic images). EGFR p.E746_A750del (c.2235_2249del15) was confirmed (Figure 1B). IPHC was conducted in left thoracic cavity in October 2009, followed by 4 cycles of systemic gemcitabine plus cisplatin treatment. Lung CT detected complete regression of mediastinal lymph nodes, significant regression in nodule in upper left lobe (Figure 1A, lower left panel, highlighted with blue arrow-head), and disappearance of left pleural effusion (Figure 1A, lower left panel, highlighted with red arrow-head). The patient was in good health for four years until lung CT revealed tumor nodules in left lung in 2013 and the patient began taking icotinib, an EGFR inhibitor approved for clinical usage by China Food and Drug Administration (CFDA) [17, 18]. More patients respond to IPHC-CT treatment was shown in Figure 1C (history of the patients was listed in supplementary file).

After checking their clinical records, we noticed that patients positive for EGFR kinase domain mutations were the population most likely to be benefited from the IPHC-CT treatment. We then plotted patients' survival using Kaplan–Meier curve in our records. Consistent with earlier reports [14–16], both EGFR mutation positive (red line) and EGFR mutation negative (blue line) cohort treated with IPHC-CT showed significantly longer overall survival over patients treated through systemic chemotherapy (black line) (left panel, Figure 1D). Importantly, significantly longer overall survival was seen in EGFR kinase domain mutation positive patients over negative patients in response to IPHC-CT (Figure 1D). Summary of patients' demographic information is shown in Figure 1E.

### Hyperthermic chemotherapy effectively eliminated the colony-forming ability of EGFR mutant lung cancer cells

**Figure 2:**
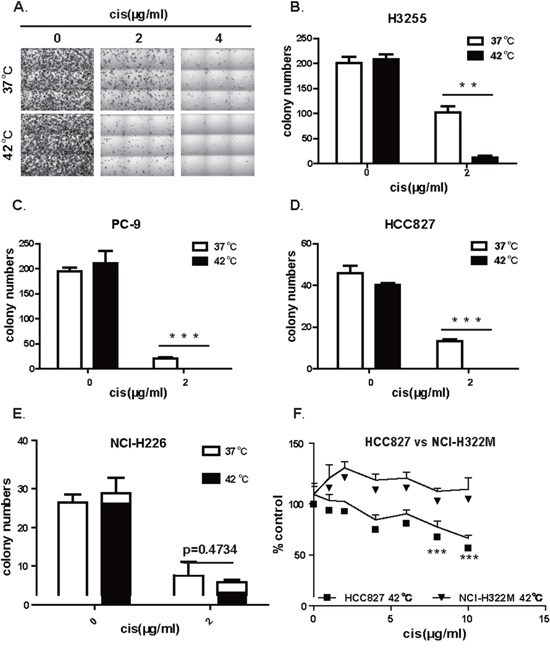
Hyperthermia synergizes with cisplatin in eliminating the ability of colony formation in lung cancer cell positive for EGFR mutation **A.** Representative images of colonies formed by H3255 (L858R) treated at 37 or 42°C in the presence of 0, 2, or 4 (μg/mL of cisplatin. **B.** Statistics of colony formation result of H3255 (L858R) showed that hyperthermia sensitized H3255 cell to cisplatin treatment. **C.** Statistics of colony formation result of PC-9 (exon19 deletion) showed that hyperthermia sensitized PC-9 cell to cisplatin treatment. **D.** Statistics of colony formation result of HCC827 (exon19 deletion) showed that hyperthermia sensitized HCC827 cell to cisplatin treatment. **E.** Statistics of colony formation result of NCI-H226 with wildtype EGFR showed no difference between HC and NC treatment. **F.** Comparison of IPHC killing efficiency between HCC827 (exon19 deletion) and NCI-H322M (wild-type EGFR) cell lines under HC treatment. Error bars, mean ± SD; **p < 0.01, ***p < 0.001, unpaired two-tailed *t*-test; *n* = 3 biological replicates (B, C, D, E, F).

To further study the mechanism underlying the IPHC-CT therapy, we tested whether Hyperthermic Chemotherapy affect EGFR mutant cells in a cell autonomous manner. To recapitulate therapy in an *in vitro* setting, we assayed the impact of Hyperthermic Chemotherapy on the colony formation ability of EGFR mutant NSCLC cell lines (H3255, PC-9 and HCC827). The cells were treated at 37°C (mock), 37°C wtih cisplatin (normothermic chemotherapy, NC), 42°C alone (hyperthermic, H), and 42°C with cisplatin (hyperthermic chemotherapy, HC) for 2 hours, and then incubated at 37°C for 10 to 15 days in fresh media without cisplatin until colonies were visible. H3255, a cell line derived from an NSCLC patient harboring the L858R mutation, was highly sensitive to HC treatment compared with hyperthermic monotherapy at 42°C or cisplatin monotherapy (Figure 2A). The statistics revealed a significantly higher efficacy of hyperthermic chemotherapy in comparison to hyperthermic therapy or chemotherapy alone (Figure 2B). We tested the *in vitro* colony formation of two other cell lines, PC-9 and HCC827, both of which are positive for the exon 19 deletion mutation, the most frequently detected EGFR mutation in NSCLC patients. Similarly, we found that these cell lines were also sensitive to HC (Figure 2C, 2D). To tell whether dramatic HC response is associated with EGFR mutational status, we also conducted colony formation assay on NCI-H226 cells, an EGFR wild-type lung cancer cell line (Figure 2E) and found no significant difference between the NC and HC treated groups. In all, our *in vitro* data faithfully recapitulated our clinical observations that IPHC is highly effective in treating NSCLC patient positive for EGFR kinase domain mutations.

The above results suggested that EGFR mutation positive lung cancer cell lines were sensitive to HC treatment. To further confirm our conclusion, we compared cytotoxicity of hyperthermic chemotherapy on EGFR mutation positive cell lines side by side with lung cancer cell lines negative for EGFR mutation. Consistent with earlier reports, we found that chemotherapy treatment at 42°C conferred significantly higher toxicity on EGFR mutation positive cell line HCC827 than on EGFR mutation negative cell line NCI-H322M (Figure 2F). This is confirmed on another pair of lung cancer cell lines: EGFR mutation positive H1650 cell and EGFR wildtype NCI-H226 cell (Supplementary Figure S1).

### Hyperthermic chemotherapy induced apoptosis in EGFR mutant cells

**Figure 3:**
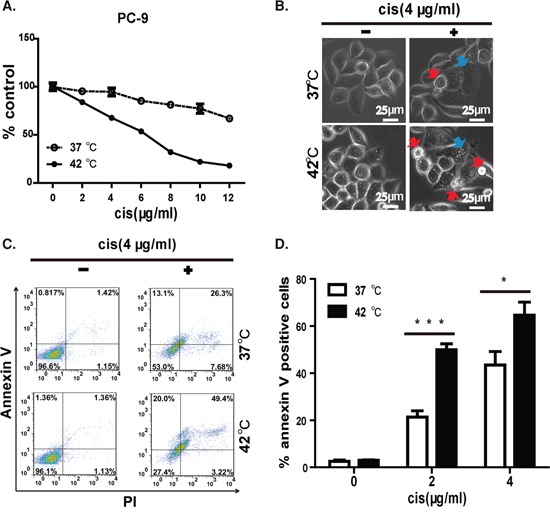
Hyperthermia synergizes cisplatin to induce apoptosis in EGFR mutant lung cancer cell line **A.** IC50 of cisplatin in killing PC-9 cell is lower at 42°C than at 37°C. **B.** Morphology of PC-9 cell under 37 or 42°C in the presence of 4 (μg/mL of cisplatin. Apoptotic cells are highlighted in red arrow and cells undergoing apoptosis are highlighted in blue arrow. **C.** Representative FACS data showed hyperthermia synergizes with cisplatin in inducing apoptosis on PC-9 cells. **D.** Statistics of FACS data, error bars, mean ± SD; *p < 0.05, ***p < 0.001, unpaired two-tailed *t*-test, *n* = 3 biological replicates.

The high efficacy of HC in inhibiting cell growth and colony formation suggests an active killing of tumor cells. We then measured the inhibitory concentration (IC) 50 values of cisplatin under hyperthermic and normothermic conditions. As shown in Figure 3A, the IC50 for the PC-9 cell line was 6 μg/ml (∼20 μM) under hyperthermia but was substantially higher under normothermic conditions. Our data, therefore, suggest the synergistic effect between hyperthermia and cisplatin in killing PC-9 cells. We checked carefully the morphology of the cells treated under these conditions, and found that cells were healthy under mock and 42°C treated cells. In contrast, cisplatin treated cells showed shrinkage of the cell and the nucleus. We also noticed condensation of nuclear chromatin into sharply delineated masses that become marginated against the nuclear membranes, which is typical of apoptotic cells. Strikingly, we detected much more apoptotic cells treated with cisplatin under 42°C (Figure 3B).

We next sought to further confirm that apoptosis was involved in HC-induced cell death. PI/annexin V staining clearly detected late-stage apoptosis in 49.4% of HC-treated PC-9 cells, which was significantly more than that observed with cisplatin monotherapy (26.3%). In contrast, hyperthermia monotherapy for 2 h had no significant influence on cell morphology or survival. This result clearly showed that hyperthermia synergized with cisplatin to induce apoptosis (Figure 3C). Moreover, this difference was statistically significant at 2 and 4 μg/ml of cisplatin tested (Figure 3D).

### Hyperthermia promoted higher cisplatin accumulation in EGFR kinase domain mutation positive cells and synergized with cisplatin to inhibit EGFR signaling

**Figure 4:**
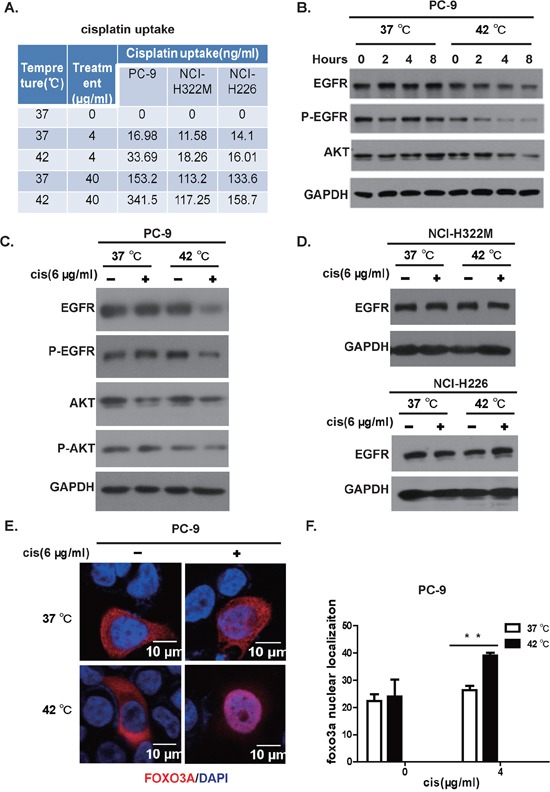
Hyperthermia synergizes cisplatin in killing lung cancer cell positive for EGFR mutation **A.** Hyperthermia enhances accumulation of cisplatin within the cell. PC-9, NCI-H322M, NCI-H226 cells were incubated at 37 or 42°C in the presence of 0, 4, 40 (μg/mL of cisplatin. Intracellular cisplatin concentration was determined through inductively coupled plasma-mass spectrometry (ICP-MS). **B.** Hyperthermia downregulates the EGFR protein level in a time-dependent manner. **C.** Western boltting analysis of PC-9 cells in response to mock, NC, H, HC treatment. **D.** Western blotting of EGFR protein levels on NCI-H322M (upper panel) and NCI-H226 (lower panel) cells under mock, NC, H, and HC treatment. **E.** FoxO3 translocated to nuclei in response to hyperthermia/cisplatin combinational therapy in PC-9 cells (red: FoxO3 immunostaining; blue: Dapi for nuclear staining). **F.** Statistics of nuclear FOXO3 protein signals determined by immunostaining, error bars, mean ± SD; **p < 0.01, unpaired two-tailed *t*-test, *n* = 3 biological replicates.

Our cytotoxicity experiment on cell lines showed that hyperthermia and cisplatin synergistically killed EGFR kinase domain mutation positive cancer cells. In order to study the mechanism underlying this synergy, we tested whether hyperthermia leads to accumulation of cisplatin in EGFR mutation positive cells. We therefore treated EGFR mutant PC-9 cell and EGFR wildtype NCI-H322M and NCI-H226 cells with 4 and 40 μg/ml of cisplatin at 37 and 42°C for 2 hours and washed the cells immediately with PBS and homogenize the cells for mass spectrometry to assay the concentration of cisplatin accumulated inside cells. Consistent with earlier report, we found higher intracellular cisplatin accumulation when treated at 42°C [19, 20]. Strikingly, we found that 42°C treatment resulted in almost double the amount of intracellular cisplatin compared to 37°C treated group at both concentrations in EGFR mutation positive cell line PC-9. In contrast, the EGFR wildtype cell lines NCI-H322M and NCI-H226 showed only slight increase (Figure 4A).

Hyperthermia has been reported to cause cellular protein degradation [21]. Chemotherapy has also been shown to cause protein degradation through ubiquitination dependent pathway. We wonder whether a synergy in degrading EGFR protein exists for hyperthermia and cisplatin. In our hand, we didn't see a dramatic downregulation of EGFR by cisplatin for up to 8 hours at 37°C in PC-9 cells. In striking contrast, cisplatin dramatically downregulated the protein level of EGFR in these cells at 42°C in a time-dependent manner (Figure 4B). This trend was even more obvious at phospho-EGFR level (Figure 4B).

To recapitulate the clinical setting, we treated the cell line with hyperthermia and cisplatin at doses used in clinical IPHC-CT. In our system, we found that 42°C exposure for 2 hours did not significantly affect levels of total EGFR protein in PC-9 cells, neither did cisplatin treatment (Figure 4C). In striking contrast, we found that EGFR was drastically downregulated in HC-treated PC-9 cells (Figure 4C). To show whether HC induced EGFR degradation was EGFR mutation status associated, we repeated this experiment on EGFR wildtype NCI-H322M and NCI-H226 cells. As expected, the EGFR levels showed no significant difference between HC and NC treated groups (Figure 4D). Phosphorylation of EGFR plays a critical role in eliciting downstream signaling. Consistently, we also observed that phospho-EGFR was drastically decreased in HC-treated PC-9 cells compared with H or NC or mock-treated cells (Figure 4C). The phospho-EGFR of NCI-H322M and NCI-H226 cells was only detectable under extremely long exposure, consistent with the fact that growth of these two cells rarely dependent on EGFR signals. We detected no significant difference between HC-treated group and H-, NC- or Mock-treated groups (data not shown). Accordingly, we found that Akt phosphorylation was downregulated in the HC-treated PC-9 cells, which is consistent with the fact that Akt is a critical downstream signaling partner of EGFR. FoxO3 shuttles between the nuclei and the cytoplasm in response to phosphorylation by Akt. Consistently, we found that significantly more HC-treated cells showed nuclear staining of FoxO3, as assessed through immunofluorescent staining, than mono- or mock-treated cells (Figure 4E, statistics shown in Figure 4F).

### Hyperthermia and cisplatin synergistically activated the apoptotic machinery

**Figure 5:**
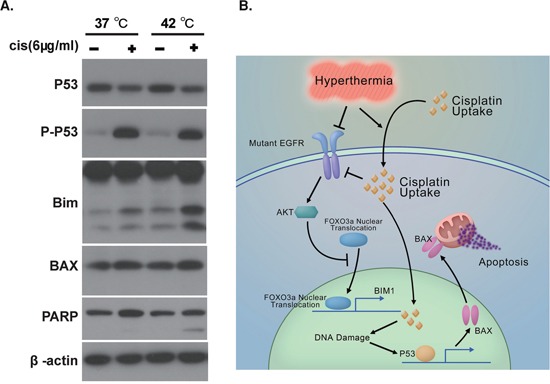
Hyperthermia and Cisplatin synergistically activated the apoptotic machinery **A.** Western analysis of apoptosis signals in PC-9 cells in response to mock, NC, H, and HC treatment. **B.** Schematic figure summarizing mechanism underlying hyperthermic chemotherapy induced apoptosis.

Inhibition of EGFR signaling leads to apoptosis of EGFR mutation positive tumor cells, a phenomenon termed oncogene addiction [22]. We then explored the molecular events underlying the activation of apoptosis in lung cancer cells treated with hyperthermic chemotherapy. We found that expression of Bim-1, the transcriptional target of FoxO3, was upregulated in HC-treated PC-9 cells, but not in cells under other conditions (Figure 5A).

The main target of intracellular target of cisplatin is DNA. Double- or single-strand break of DNA leads to activation of p53 through ATR and ATM. We found that p53 was highly activated in PC-9 cells after exposure to 6 μg/ml cisplatin at nomothermic and hyperthermic conditions (Figure 5A). Accordingly, we detected an upregulation of Bax, a transcriptional target of p53, in cells harboring activated p53 (Figure 5A).

BIM-1 can interact with BCL-2 to allow the release of BAX, which in turn drives cytochrome c release from the mitochondrion to elicit apoptosis. Accordingly, we detected significant PARP cleavage in HC-treated PC-9 cells but not in mono- or mock-treated cells (Figure 5A).

We have summarized our proposed mechanism in Figure 5B. Hyperthermia promotes accumulation of intracellular cisplatin in EGFR mutation positive lung cancer cells. Enhanced level of intracellular cisplatin causes genomic DNA damages, thus activating p53 to induce apoptotic effecter proteins such as Bax. In addition, cisplatin and hyperthermia synergize to downregulate the protein level of EGFR. Akt phosphorylation is therefore inhibited, which in turn causes the translocation of FoxO3 to the nuclei, where it induces Bim-1 expression. Bim-1 then activates Bax to damage the mitochondria and elicit apoptosis.

## DISCUSSION

In our current work, we clearly showed that IPHC-CT was effective against lung cancers positive for EGFR kinase domain mutations. We therefore suggest that IPHC-CT should be considered on lung cancer patients positive for EGFR kinase domain mutation.

Chemotherapy is the mainstay of treatment for patients with advanced lung cancer. Unfortunately, the prognosis for these patients remains disappointingly poor. Interestingly, EGFR mutation has been found to be a favorable prognosis marker in chemotherapy treated patients [23–25]. On the other hand, EGFR has been reported to cause resistance to chemotherapy through multiple mechanisms: activation of the mTORC2-NF-κB signaling pathway [26]; activation of the Jak-Stat3 pathway [27]; upregulation of anti-apoptotic effecter proteins [28]; and translocation to the nucleus where it participates in DNA repair through interacting with DNA-PK [29] and ERCC1 [30]. In our current report, clinical cases have shown that EGFR kinase domain mutation positive lung cancers respond dramatically to combinational treatment of hyperthermia and cisplatin. Biochemical analysis consistently revealed mechanism underlying the cytotoxicity of IPHC-CT to tumor cells.

Although Erlotinib and Geffitinib have shown great success in shrinking kinase domain mutant EGFR positive lung cancers, resistance to EGFR-targeted therapy is almost an inevitable obstacle in the clinic. Therefore, effective alternatives are desperately needed in the clinic for treating EGFR mutation-positive lung cancer patients.

In our current work, we found that the hyperthermic conditions used in clinical practice for treating lung cancer patients by IPHC does not significantly affect the EGFR protein level in lung cancer cell lines. A similar effect was found for cisplatin treatment. Strikingly, we found that the combination of cisplatin and hyperthermia significantly decreased the EGFR protein level in EGFR mutant-driven lung cancer cells. This synergistic effect can be explained through the following possibilities: 1) the local cisplatin concentration during IPHC was high. As reported by Koga, 16.7 μg/g of free cisplatin was detected in pleural metastatic tissues [14]; and 2) increased cisplatin accumulation in tumor cells under hyperthermic condition as we saw in EGFR mutation positive cell lines.

Of note, while we observed a drastic decrease in EGFR phosphorylation in the cells treated with hyperthermic chemotherapy, more importantly, we observed decreased EGFR protein level. Recently, kinase-independent activity of EGFR has been recognized, the first hint of which was from genetically modified mice. Specifically, EGFR-knockout mice do not survive to adulthood, while EGFR kinase-dead mutant mice were viable, although presenting with abnormalities in the skin and eye [31, 32] (for review, see [33]). Drastic downregulation of EGFR protein level by IPHC is therefore expected to elicit stronger cytotoxicity than EGFR kinase inhibition alone.

Considering that IPHC and TKIs target EGFR through different mechanisms, these two therapies are therefore not mutually exclusive. As such, intensive research should be carried out to evaluate the synergistic effect of TKIs and IPHC.

Our work clearly shows that IPHC-CT is effective against lung cancer patients harboring mutant EGFR. We, therefore, suggest that IPHC should be considered for this subset of patients.

## MATERIALS AND METHODS

### Ethics statement

This study was approved by Beijing Tongren Hospital, Capital Medical University, Beijing, China and the Institutional Committee at the National Institute of Biological Sciences, Beijing (NIBS). All experiments were performed in accordance with relevant guidelines and regulations. Written consent was obtained from every patient who donated tissues.

### Surgical procedure

IPHC was routinely conducted in our hospital as we reported earlier [34].

### Mutational analysis

EGFR mutational status was checked through high-performance liquid chromatography by Surexam Biotechnology Co., Ltd (Guangdong, China) from 10–20 pieces of unbaked slices from paraffin-embedded tumor specimens or formalin fixed biopsy derived from surgery. Sanger sequencing was conducted to verify the mutation on patients if extra tumor tissue was available. Procedure for extracting DNA and PCR amplification of exon 18–21 are listed in Supplementary Methods.

### Cell culture

The human lung cancer cell lines PC-9, HCC827, H3255, H1650, NCI-H322M, and NCI-H226 were obtained from the American Type Culture Collection (ATCC) (Manassas, VA, USA). PC-9, HCC827, H1650, NCI-H322M and NCI-H226 were cultured in RPMI 1640 medium (SH3080901b, Hyclone) supplemented with 10% FBS (10099141, Gibco), L-glutamine (25030-081, Invitrogen), and 100 U/ml penicillin and streptomycin (SV3001001, Hyclone). H3255 was cultured in ACL-4 medium (serum-free) supplemented with 0.02 mg/ml insulin (I-2797, Sigma-Aldrich), 0.01 mg/ml transferrin (T-5391, Sigma-Aldrich), 25 nM sodium selenite (S-9133, Sigma-Aldrich), 50 nM hydrocortisone (H-6909, Sigma-Aldrich), 1 ng/ml epidermal growth factor (E-9644, Sigma-Aldrich), 0.01 mM ethanolamine (E-0135, Sigma-Aldrich), 0.01 mM phosphorylethanolamine (P-0503, Sigma-Aldrich), 100 pM triiodothyronine (T-6397, Sigma-Aldrich), 0.5% (w/v) bovine serum albumin (A-9418, Sigma-Aldrich), 10 mM HEPES (15630-080, Gibco), 0.5 mM sodium pyruvate (11360-070, Gibco), and 2 mM L-glutamine (G-5763, Sigma-Aldrich).

### Colony formation assay

Cells were plated at a density of 500 cells/well in triplicate in 6-well plates and incubated for 24 h before treatment. Cisplatin (H37021758, Qilu Pharmaceutical Co., Ltd, Shandong, China) was freshly dissolved in 0.9% NaCl to a concentration of 0.8 mg/ml for use. After treatment with cisplatin at 42°C or 37°C for 2 h, the spent media was replaced with 2.0 mL fresh media, and the cells were cultured for 10 days at 37°C. The media were removed, and the cells were washed 3 times with PBS (SH3025601, Hyclone). Cells were fixed with 70% ethanol for 15 min, stained with 2% crystal violet (C3886, Sigma-Aldrich) for 15 min at room temperature, washed with PBS to remove the remaining dye, and air dried. The images were scanned by Nikon A1-R, and the area of the cell colonies was analyzed with Image-Pro Plus software. The cutoffs used were H3255 > 12000 μm^2^, PC-9 > 1000 μm^2^, and HCC827 > 4000 μm^2^.

### Cell viability assay

Cells were plated in 384-well plates at a density of 3000 cells/well 24 h before treatment. The cells were then treated with 0, 2, 4, 6, 8, 10, or 12 μg/mL cisplatin for 2 h at 37 or 42°C. The media was then replaced with 50 μl fresh media, and the cells were cultured for an additional 48 h, at which time, cell viability was measured using the Cell Titer Glo luminescent assay (G7572, Promega).

### PI and Annexin V staining

PC-9 cells were plated in 6-well plates at 50% confluence 24 h before treatment. After hyperthermia and cisplatin treatment for 2 h, the spent media were replaced with 2.0 mL of fresh media, and the cells were cultured for 48 h at 37°C. The cells were harvested with 0.25% trypsin (25200–056, Invitrogen) and stained with Annexin V and propidium iodide (PI) using an apoptosis detection kit (APOAF, Sigma-Aldrich) according to the manufacturer's instructions.

### Western blot (WB) assays

In total, 1.2×10^6^ cells/10-cm dish were plated 24 h before treatment. After hyperthermia and cisplatin treatment for 2 h, the spent media was replaced with 10 mL fresh media, and the cells were cultured for 24 h at 37°C. The floating apoptotic cells were then collected through centrifugation, and the attached cells were collected through trypsinization. The combined cell pellets were homogenized with lysis buffer (50 mM Tris pH 7.4, 150 mM NaCl, 1 mM EDTA, 1% Triton X-100, 10% glycerol) on ice for 45 min. The following antibodies were used for WB: EGFR (4267, Cell Signaling Technology); EGFR-Y1068 (3777, Cell Signaling Technology); AKT (9272, Cell Signaling Technology); AKT-S473 (4060, Cell Signaling Technology); P53 (2527, Cell Signaling Technology); P53-S15 (9286, Cell Signaling Technology);; PARP (9542, Cell Signaling Technology); Bim-1 (2933, Cell Signaling Technology); β-actin (A5316, Sigma-Aldrich); and Bax (2772, Cell Signaling Technology).

### Immunofluorescent staining

PC-9 cells were plated in 6 well plates with steriled coverslips and transfected with 3 μg/well of Flag-FoxO3a vectors 24 h before treatment. After hyperthermia and cisplatin treatment for 2 h, the spent media were replaced with 2.0 mL of fresh media, and the cells were cultured for 18 h at 37°C. Cells on the coverslips were fixed with 4% PFA for 25 minutes at room temperature and washed four times with PBS. Next, the cells were blocked with 5% normal goat serum containing 0.3M glycine 1 h at room temperature. After blocking, anti-Flag antibody (F1804, Sigma) was incubated overnight at 4°C, followed with four times washing and incubation of secondary antibodies. After wash, nuclear dye DAPI was added to each well and stained for 3–5 minutes at room temperature, then washed away and mounted with 70% glycerol+30%PBS.

### Statistic analysis

Student T test (Microsoft Excel (Microsoft, Redmond, WA, USA)) was used to compare two groups, *p* < 0.5(*) was considered as statistically significant, *p* < 0.01(**) was considered as very significant and *p* < 0.001(***) was considered as extremely significant.

## SUPPLEMENTARY FIGURES AND TABLES


