# Correction: EGFR kinase domain mutation positive lung cancers are sensitive to intrapleural perfusion with hyperthermic chemotherapy (IPHC) complete treatment

**DOI:** 10.18632/oncotarget.26255

**Published:** 2018-10-16

**Authors:** Hongjuan Zhang, Cheng Zhan, Ji Ke, Zhiqiang Xue, Aiqun Zhang, Kaifeng Xu, Zhirong Shen, Lei Yu, Liang Chen

**Affiliations:** ^1^ School of Life Science, Tsinghua University, Beijing 100084, China; ^2^ National Institute of Biological Sciences, Beijing 102206, China; ^3^ Beijing Tongren Hospital, Capital Medical University, Beijing 100730, China; ^4^ The General Hospital of People’s Liberation Army (301 Hospital), Beijing 100853, China; ^5^ Peking Union Medical College Hospital, Beijing 100730, China; ^6^ Collaborative Innovation Center of Systems Biomedicine, Shanghai Jiao Tong University School of Medicine, Shanghai 200240, China; ^7^ National Institute of Biological Sciences, Collaborative Innovation Center for Cancer Medicine, Beijing, 102206, China

**This article has been corrected:** The correct Acknowledgment information is given below:

**ACKNOWLEDGMENT**

This work was funded by National Natural Science Foundation of China grant 81472606, Chinese Ministry of Science and Technology grant (973 grant) 2011CB812401, and the Beijing Municipal Government.

Original article: Oncotarget. 2016; 7:3367-3378. https://doi.org/10.18632/oncotarget.6491

