# Supplementary material for: EGFR kinase domain mutation positive lung cancers are sensitive to intrapleural perfusion with hyperthermic chemotherapy (IPHC) complete treatment
Source: Oncotarget. 2015 Dec 8;7(3):3367–78. doi: 10.18632/oncotarget.6491 (PMC4823112; doi:10.18632/oncotarget.6491)
Supplement: Supplementary file 1 [file oncotarget-07-3367-s001.pdf]

SUPPLEMENTARY PATIENTS' SUMMARY

1. Patient# 528908, female, 67 years old

In July 2011, patient was diagnosed of MIa stage lung adneocarcinoma in the middle lobe of right lung, with moderate amount of pleural effusion in the right thoracic cavity and multiple metastases sites on the right chest wall and pleura (Figure 1C, a). EGFR E746_A750del(K745:AAG) mutation was detected by Surexam Biotechnology Co., Ltd (Guangdong, China) in the needle biopsy. IPHC was conducted in the right thoracic cavity, followed by 6 cycles of chemotherapy with gemcitabine and cisplatin. CT examination in November 2011 revealed shrinkage of both primary tumor and metastatic nodule and the right thoracic cavity (Figure 1C, b). Tumor volume further shrinked in May 2012. The patient was further treated by 6 cycles of taxol and cisplatin treatment. In November 2012, CT showed that tumor volume continued to shrink (Figure 1C, c). The patient started to take Icotinib, an EGFR inhibitor approved for clinical usage by China Food and Drug Administration (CFDA). PET-CT conducted in February 2013 showed that the tumor lesion was inactive. Till the paper preparation, the patient was still alive.

2. Patient# 537050, female, 68 years old

In February 2012, CT detected lung adenocarcinoma in the upper lobe of left lung accompanied by moderate amount of plural effusion in the left thoracic cavity, metastases sites on the plura and left chest wall and mediastinal lymph node enlargement (Figure 1C, d). EGFR E746_A750del(K745:AAG) mutation was detected Surexam Biotechnology Co., Ltd (Guangdong, China) in the needle biopsy. IPHC followed by 6 cycles of gemcitabine and cisplatin was conducted. In June 2012, CT scanning revealed shrinkage of both primary tumor and metastases nodules and enlargement of mediastinal lymph node (Figure 1C, e). The patients refused to be treated through chemotherapy and started to take Icotinib. The most recent CT examination showed that tumor volume continue shrinking in February 2014 (Figure 1C, f). At the time of the manuscript preparation, the patient was still alive.

3. Patient# 557902, female, 68 years old

In March 2013, CT scanning revealed tumor nodule on the upper lobe of left lung. Moderate amount of plural effusion and metastatic lesions to the chest wall and plura was noticed (Figure 1C, g). Pathological examination confirmed lung adenocarcinoma. EGFR E746_A750del (K745:AAG) mutation was detected Surexam Biotechnology Co., Ltd (Guangdong, China) in the needle biopsy. IPHC was conducted followed by 4 cycles of gemcitabine and cisplatin adjuvant therapy. In July 2013, CT detected dramatic tumor shrinkage, with only trace of lesions left (Figure1C, h). Due to the poor health condition, Icontinib was choosed instead of chemotherapy. In October 2013, the primary tumor foci disappeared. In March 2014, CT revealed pleural calcification but not tumor foci (Figure 1C, i). At the time of preparation of the manuscript, the patient was still alive.

4. Patient# 530770, male, 83 years old

In September 2011, tumor nodule on the upper lobe of right lung was detected with moderate plural effusion and metastases to the right chest wall and plura (Figure 1C, j). The tumor was diagnosed as lung adneocarcinoma. EGFR L858R(CTG > CGG) mutation was detected Surexam Biotechnology Co., Ltd (Guangdong, China) in the tumor biopsy. IPHC was conducted, followed by Icotinib because of poor health condition due to old age. In January 2012, dramatic regression in both primary and the metastatic sites were noticed in CT scan (Figure 1C, k). In June 2012, tumor continued shrinking. CT examinations in February and August revealed that the primary tumor foci was enlarged with multiple new metastatic sites (Figure 1C, l). The patient died on 23th October 2013.

SUPPLEMENTARY MATERIAL AND METHODS

PCR amplification of EGFR exons for Sanger sequencing to verify mutations

DNA was extracted with the QIAamp DNA FFPE Tissue Kit (56404, Qiagen) according to the manufacturer's instructions. Nested PCR was used to amplify the 18–21 exons of EGFR with the primers listed in the supplementary table (O=outer, I=inner).

E18-F-O GGCACTGCTTTCCAGCATGGTG
E18-R-OTATACAGCTTGCAAGGACTCTGG
E18-F-I actgtaaaacgacggccagt GACCCTTGTCTCTGTG TTCTTGT
E18-R-I accaggaaacagctatgacc CTCCCCACCAGACCAT GAGAG
E19-F-O GTGCATCGCTGGTAACATCCAC
E19-R-O GGGTCTAGAGCAGAGCAGCTG
E19-F-I actgtaaaacgacggccagt CAGCATGTGGCACCA TCTCAC
E19-R-I accaggaaacagctatgaccAGAAAAGG TGGGCCT GAGGTTC
E20-F-O TCATGCGTCTTCACCTGGAAGG

E20-R-O GTGAGGATCCTGGCTCCTTATC
E20-F-I actgtaaaacgacggccagt CCTTCTGGCCACCAT GCGAAG
E20-R-I accaggaaacagctatgacc TCCCTTCCCTGATTACC TTTGC
E21-F-O CTGAATTCGGATGCAGAGCTTC
E21-R-O AAACAATACAGCTAGTGGGAAGG
E21-F-I actgtaaaacgacggccagt CCTCACAGCAGGGTC TTCTCTG
E21-R-I accaggaaacagctatgacc GTGTCAGGAAAATG CTGGCTGAC

Measurement of cisplatin uptake

PC-9 cells were treated with 4 and 40 μM cisplatin for 2 hours at 37 or 42°C. The cells were harvested immediately after treatment and washed 3 times with PBS. Intracellular cisplatin levels were analyzed by inductively coupled plasma-mass spectrometry (ICP-MS).

SUPPLEMENTARY FIGURE

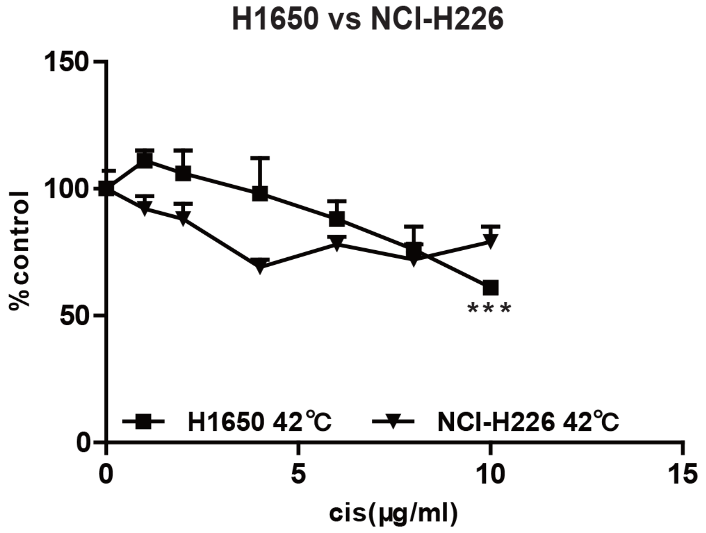

Supplementary Figure S1: Hyperthermic chemotherapy confers higher toxicity on EGFR mutation positive cells than on negative cells. Comparison of IPHC killing efficiency between H1650 (exon19 deletion) and NCI-H226 (wild-type EGFR) cell lines.
